# Regional Fluctuation in the Functional Consequence of LINE-1 Insertion in the *Mitf* Gene: The Black Spotting Phenotype Arisen from the *Mitf^mi-bw^* Mouse Lacking Melanocytes

**DOI:** 10.1371/journal.pone.0150228

**Published:** 2016-03-01

**Authors:** Kazuhisa Takeda, Hiroki Hozumi, Koji Ohba, Hiroaki Yamamoto, Shigeki Shibahara

**Affiliations:** 1 Department of Molecular Biology and Applied Physiology, Tohoku University School of Medicine, Sendai, Miyagi 980–8575, Japan; 2 Faculty of Bioscience, Nagahama Institute of Bio-Science and Technology, Nagahama, Shiga 526–0829, Japan; Rutgers University, UNITED STATES

## Abstract

Microphthalmia-associated transcription factor (Mitf) is a key regulator for differentiation of melanoblasts, precursors to melanocytes. The mouse homozygous for the *black-eyed white* (*Mitf*^*mi-bw*^) allele is characterized by the white-coat color and deafness with black eyes due to the lack of melanocytes. The *Mitf*^*mi-bw*^ allele carries LINE-1, a retrotransposable element, which results in the Mitf deficiency. Here, we have established the black spotting mouse that was spontaneously arisen from the homozygous *Mitf*^*mi-bw*^ mouse lacking melanocytes. The black spotting mouse shows multiple black patches on the white coat, with age-related graying. Importantly, each black patch also contains hair follicles lacking melanocytes, whereas the white-coat area completely lacks melanocytes. RT-PCR analyses of the pigmented patches confirmed that the LINE-1 insertion is retained in the *Mitf* gene of the black spotting mouse, thereby excluding the possibility of the somatic reversion of the *Mitf*^*mi-bw*^ allele. The immunohistochemical analysis revealed that the staining intensity for beta-catenin was noticeably lower in hair follicles lacking melanocytes of the homozygous *Mitf*^*mi-bw*^ mouse and the black spotting mouse, compared to the control mouse. In contrast, the staining intensity for beta-catenin and cyclin D1 was higher in keratinocytes of the black spotting mouse, compared to keratinocytes of the control mouse and the *Mitf*^*mi-bw*^ mouse. Moreover, the keratinocyte layer appears thicker in the *Mitf*^*mi-bw*^ mouse, with the overexpression of Ki-67, a marker for cell proliferation. We also show that the presumptive black spots are formed by embryonic day 15.5. Thus, the black spotting mouse provides the unique model to explore the molecular basis for the survival and death of developing melanoblasts and melanocyte stem cells in the epidermis. These results indicate that follicular melanocytes are responsible for maintaining the epidermal homeostasis; namely, the present study has provided evidence for the link between melanocyte development and the epidermal microenvironment.

## Introduction

Long interspersed element-1 (LINE-1 or L1) is a retrotransposable element that could cause various types of diseases [1]. The insertion of LINE-1 in a certain gene may result in the impaired expression of an affected gene and/or the altered function of an affected gene product. Incidentally, LINE-1 is present in intron 3 of the *microphthalmia-associated transcription factor* (*Mitf*) gene of the black-eyed white (*Mitf*^*mi-bw*^) mouse [2]. Mitf has been established as an essential regulator for the development of melanoblasts, precursors to melanocytes [3–5]. The mouse homozygous for the *Mitf*^*mi-bw*^ allele shows a complete black-eyed white phenotype with severe hearing loss but without apparent ocular abnormalities [2, 6], the phenotype of which is due to the lack of melanocytes.

Melanoblasts migrate from the neural crest during fetal development to distribute mainly to the skin, eye (iris and choroid), and inner ear, thereby contributing to pigmentation of the coat and eyes as well as to sight and hearing [7]. The LINE-1 insertion results in the aberrant splicing of *Mitf* gene transcripts [2, 8], thereby decreasing the expression level of Mitf, including Mitf-M that is preferentially expressed in melanocyte-lineage cells [9, 10]. Because the *Mitf*^*mi-bw*^ allele carries the LINE-1 in intron 3 located between exon 3 and exon 4 [2], the Mitf-M transcripts that are encoded by the downstream first exon may be more likely to undergo aberrant splicing, compared to other Mitf isoform transcripts. Thus, the *Mitf*^*mi-bw*^ mouse has provided an excellent model to study the phenotypic consequences of the Mitf-M deficiency and/or the lack of melanocytes [8, 11, 12]. Unexpectedly, however, Mitf-M mRNA is expressed in the wild-type mouse brain as well as in the homozygous *Mitf*^*mi-bw*^ mouse brain [8, 11]. Subsequently, we have shown the Mitf-M expression in the projection neurons of the olfactory bulb [13]. These results suggest that Mitf-M expression may be regulated in neurons by a mechanism distinct from that in melanocyte-lineage cells. Moreover, the influence of the LINE-1 insertion may be different between neurons and melanocyte-lineage cells.

In the present study, we aimed to explore the functional consequence of the LINE-1 insertion using the homozygous *Mitf*^*mi-bw*^ mouse skin. In this context, Silvers [7] described a black spotting phenotype, arisen from the black-eyed white mouse on the C3H strain. We also obtained the *Mitf*^*mi-bw*^ mouse with black spots that was spontaneously arisen from the homozygous *Mitf*^*mi-bw*^ mouse on a mixed background of C3H and C57BL/6J (C3;B6-*Mitf*^*mi-bw*^*/Mitf*^*mi-bw*^) [2, 14]. The presence of such a black spotting phenotype suggests that the functional consequence of the LINE-1 insertion may be fluctuated, depending on the cellular microenvironment. Accordingly, we have established the homozygous *Mitf*^*mi-bw*^ mouse with black spots, termed black spotting mouse. This mouse line shows black patches with a grayish tone on the white coat background, despite that the LINE-1 insertion is retained in its *Mitf* gene. Moreover, the adult black spotting mouse is characterized by age-related graying of the pigmented patches. Using the homozygous *Mitf*^*mi-bw*^ mouse and the black spotting mouse, we have provided the evidence that attenuated beta-catenin expression may be involved in the regional fluctuation in the fate of developing melanoblasts and melanocytes.

## Materials and Methods

### Mice

The mouse strain carrying the *Mitf*^*mi-bw*^ allele (C3;B6-*Mitf*^*mi-bw*^*/Mitf*^*mi-bw*^) was obtained as described previously [2, 14], and the *dopachrome tautomerase* (*Dct*)*-lacZ* transgenic mouse was kindly provided by Dr. Ian J Jackson [15]. The mouse homozygous for the *Mitf*^*mi-bw*^ allele and the *Dct-lacZ* transgene was then established on the C57BL/6J background [5]. Originally, the *Mitf*^*mi-bw*^ allele had been maintained on the C57BL/6J background [2]. However, due to the risk of extinction, the *Mitf*^*mi-bw*^ allele was rescued by crossing with C3H/He mice [2]. The *Mitf*^*mi-bw*^ mice with pigmented spots were spontaneously born in the late 1990s, during which time we had maintained the *Mitf*^*mi-bw*^ allele on the C3;B6 mixed background. Subsequently, the *Dct-lacZ* transgene was introduced into the C3;B6-*Mitf*^*mi-bw*^*/Mitf*^*mi-bw*^ mice with pigmented spots (C3;B6-*Mitf*^*mibw*^*/Mitf*^*mi-bw*^, Tg(Dct-lacZ)). The *Mitf*^*mi-bw*^ mouse line with black spots carrying the *Dct-lacZ* transgene on the C57BL/6J background (C57BL/6J-*Mitf*^*mi-bw*^*/Mitf*^*mi-bw*^, Tg(Dct-lacZ)) was established by crossing the C3;B6-*Mitf*^*mi-bw*^*/Mitf*^*mi-bw*^, Tg(Dct-lacZ) mice with pigmented spots with the C57BL/6J-*Mitf*^*mi-bw*^*/Mitf*^*mi-bw*^, Tg(Dct-lacZ mice (more than 10 generations). In the subsequent text, we use a term, the black spotting mouse, to indicate the homozygous *Mitf*^*mi-bw*^ mouse carrying the *Dct-lacZ* transgene with the black spotting phenotype, unless otherwise specified. Mice were maintained under 12 h light/12 h dark cycle at 23–25°C and were allowed free access to standard mice food and water. This study was carried out in strict accordance with the recommendations in the Guide for the Care and Use of Laboratory Animals of the National Institutes of Health. The animal experiments were performed based on the protocol approved by the Committee on the Ethics of Animal Experiments of Tohoku University Graduate School of Medicine (Permit Number: 2013MdA-251). Animals were euthanized by cervical dislocation. All efforts were made to minimize suffering.

### Antibodies

Anti-MITF polyclonal antibody was produced in rabbits using His-Tag-MITF-M as an antigen that contains a His-tag at the amino-terminus of full-length MITF-M [16]. This antibody recognizes each of MITF-M, MITF-A, MITF-C and MITF-H [16] as well as mouse Mitf [13] and chick Mitf [17, 18]. The antibody against Dct as a marker for melanocytes was purchased from Abcam (Cambridge, UK). The antibodies against beta-catenin, phosphorylated ERK1/2 (p-ERK1/2), cyclin D1, and Ki-67 were purchased from Cell Signaling Technology (Danvers, MA, USA). Non-specific normal rabbit IgG as a negative control for immunostain was purchased from DAKO (Produktionsvej, Glostrup, Denmark).

### Immunohistochemical analysis of the mouse skin

The skin samples were prepared from male control C57BL/6 mice with the *Dct-lacZ* transgene, the bw mice carrying the *Dct-lacZ* transgene, and the black spotting mice carrying the *Dct-lacZ* transgene. C57BL/6 mice were used as the wild type mouse. The mice used were 8 weeks old, unless otherwise stated. The isolated tissues were fixed with SUPER FIX (KURABO, Chuo-ku, Tokyo, Japan) at room temperature for 24 h—48 h. The tissues were paraffin-embedded and were cut into 2-μm sections for immunostaining. The tissue sections were deparaffinized, and hydrated. Unmasking of antigens was performed with 98°C water bath in Immunosaver (Wako Pure Chemical Industries) for 25 min. For blocking the endogenous peroxidase activity, the tissue sections were treated with Peroxidazed 1 (Biocare Medical, Concord, CA, USA) at room temperature for 5 min. Then, to block non-specific antibody binding, the sections were treated with Background Punisher (Biocare Medical) for 10 min at room temperature. Dilution of primary antibodies in this study was as follows: MITF, 1:400, Dct, 1:1000, beta-catenin, 1:400, cyclin D1, 1:400, p-ERK1/2, 1:400, and Ki-67, 1:400. All antibodies were diluted in Can Get Signal immunostain Solution A. The tissue sections were incubated with primary antibodies at 4°C overnight. Immunohistochemical analyses were performed with MACH4 Universal HRP-polymer (Biocare Medical) according to the manufacturer’s instructions. The tissue sections were visualized by staining with 3,3’-diaminobenzidine tetrahydrochloride (DAB) (MITF, beta-catenin, cyclin D1, p-ERK1/2 and Ki-67) or Bajoran Purple (Dct). Hematoxylin was used as counterstaining. As negative control, the tissue sections were incubated with normal rabbit IgG (DAKO), instead of a primary antibody. In case of the immunofluorescence analysis of Dct expression, the frozen section of the black patches of the black spotting mouse was used, as described previously [5].

### PCR analysis of genomic DNA

PCR was performed on 100–500 ng of genomic DNA. Cycling conditions of the PCR were as follows: initial denaturation for 2 min at 94°C, followed by a three-step profile: denaturation for 10 sec at 98°C, annealing for 30 sec at 65°C and extension for 5 min for 40 cycles. PCR amplification was performed using the following primers for LINE-1 insertion region (intron 3—exon 4 of *Mitf* gene): Fp0, 5′-GGAAAAGGGAAGTGGTAGCTTTGTG-3′ and Rp4, 5′-TTCCAGGCTGATGATGTCATCAATTACATC-3′.

### RNA isolation and RT-PCR analysis

RNA was prepared from the trunk region of each mouse embryo using TRI REAGENTTM (1 ml per 50–100 mg of tissue). The synthesized cDNA was stored at -20°C. Cycling conditions of the PCR were initial enzyme activation for 5 min at 95°C, followed by a three-step profile: denaturation for 15 sec at 95°C, annealing for 15 sec at 55°C and extension for 30–60 sec for 40 cycles. To normalize the amount of total cDNA input, the cDNA for 18S rRNA was also amplified as described above. The expression levels of Mitf-M mRNAs and 18S rRNA were detected by PCR using the following primers: Mitf-M (exon 1M—exon 3) (Fp1: 5′-ATGCTGGAAATGCTAGAATACAG-3′ and Rp3: 5′-GTTCATACCTGGGACTCACTCT-3′); Mitf-M (exon 1M—exon 7) (Fp1, 5′-ATGCTGGAAATGCTAGAATACAG-3′ and Rp7, 5′-ATGCGGTCGTTTATGTTAAATCTT-3′); and 18S rRNA (Fp, 5′-TTGACGGAAGGGCACCACCAG-3′ and Rp, 5′-GCACCACCACCCACGGAATCG-3′).

### X-gal staining of embryos from the black spotting mouse

This series of experiments was performed in parallel with the embryos from control transgenic mouse and the bw mouse [5]. Mouse embryos were washed with PBS containing 12.5 mM EDTA to remove blood components. The day of plug detection was taken to be E0.5. Timed embryos (E12.5–E17.5) were fixed with 2% paraformaldehyde by using microwave. Fixed samples were rinsed twice in PBS, and then washed in X-gal detergent (2 mM MgCl_2_, 0.05% BSA, 0.1% sodium deoxycholate, 0.02% NP-40 in 0.1 M sodium phosphate buffer pH7.3). The samples were subsequently incubated in chromogenic solution [5 mM K_3_Fe(CN)_6_, 5 mM K_4_Fe(CN)_6_, 0.085% NaCl, 0.1% X-gal in X-gal detergent] overnight at 20°C. After coloration, embryos were stored in 4% paraformaldehyde in PBS.

## Results

### Evidence for the fluctuation in the functional consequences of the LINE-1 insertion

**Fig 1:**
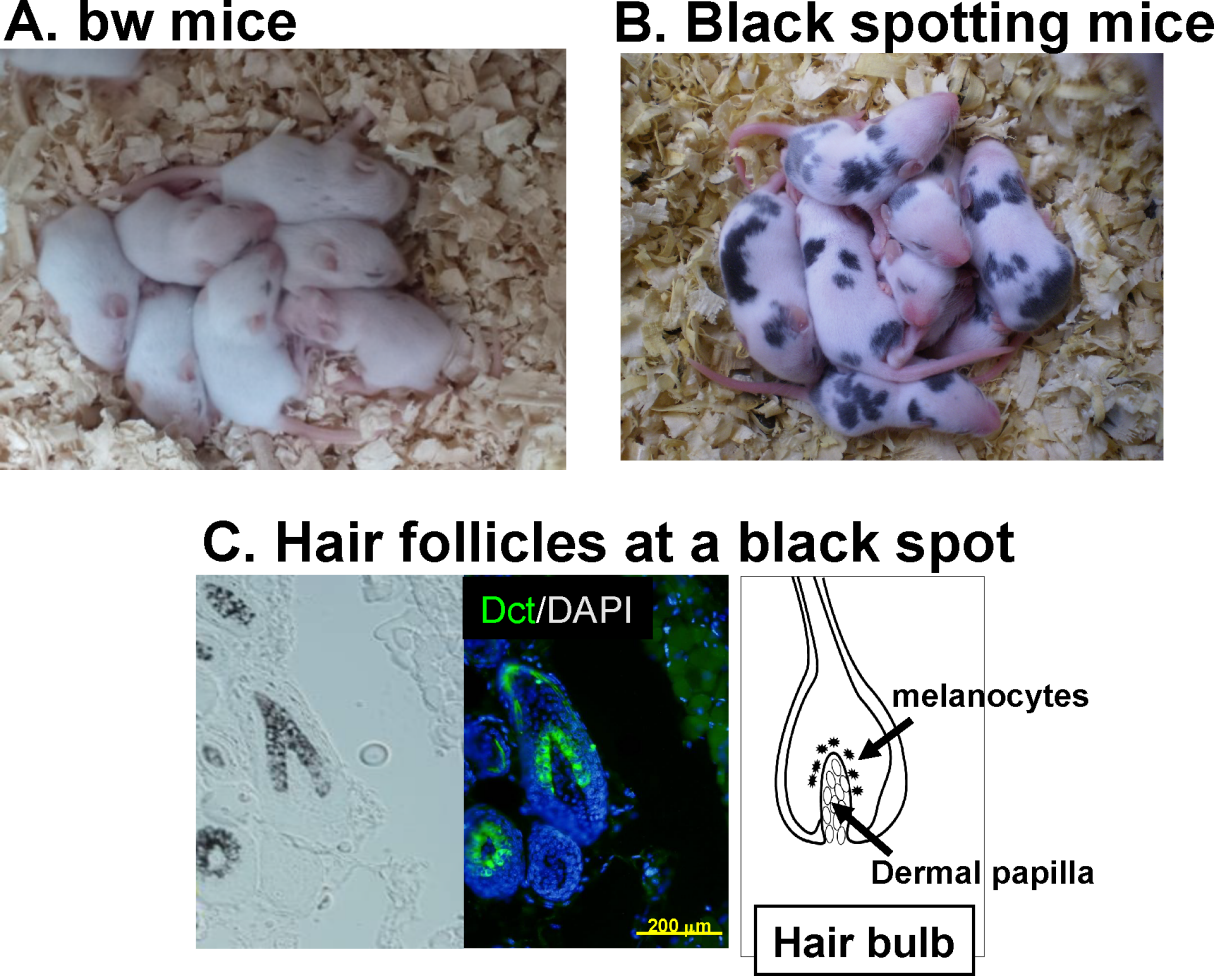
Black spotting mouse spontaneously arisen from the bw mouse. Appearance of bw mice (A) and black spotting mice (B). The newborn mice shown are 9 days old. (C) Pigmented hair follicles are present at a black patch of the black spotting mouse (30 days old). Middle image shows Dct/DAPI double staining. Expression of Dct (green signals) was detected in follicular melanocytes. Note that melanocytes are present in pigmented hair follicles. Scale bar, 200 μm.

Using the C57BL/6J mouse carrying the *Dct-lacZ* transgene, we have established the homozygous *Mitf*^*mi-bw*^ mouse carrying the *Dct-lacZ* transgene on the C57BL/6J background [5], termed bw mouse for simplicity in the present study (Fig 1A). We have also established the *Mitf*^*mi-bw*^ mouse line manifesting black spots on the C57BL/6J background carrying the *Dct-lacZ* transgene, named the black spotting mouse (Fig 1B). In contrast to the bw mouse that shows the completely white coat (Fig 1A), the black spotting mouse has black spots on the dorsal portion (Fig 1B). We then confirmed that melanocytes are present in pigmented hair follicles at a pigmented patch of the black spotting mouse, as judged by the presence of melanin pigments and Dct expression (Fig 1C). The distribution of melanocytes in a pigmented hair follicle is similar to that seen in the wild-type C57BL/6J mouse [5], although the number of pigmented hair follicles is limited in a given black patch of the black spotting mouse, as detailed below.

### Features of black patches of the black spotting mouse

**Fig 2:**
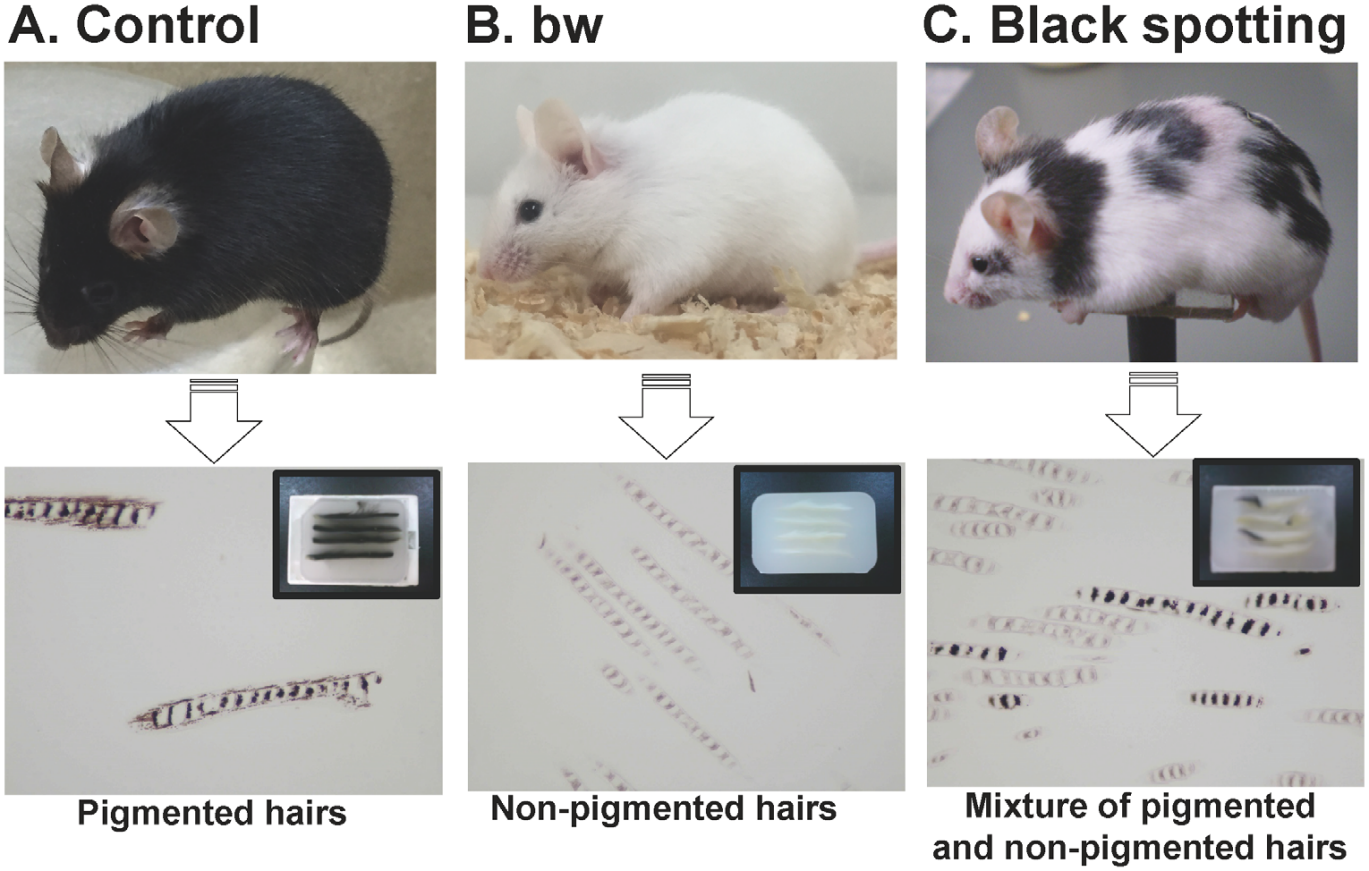
Black spotting mouse shows pigmented patches with a grayish tone. Top panels show a control lacZ transgenic mouse on the C57BL/6 background (A), the bw mouse with the black-eyed white phenotype (B), and the black spotting mouse with the black-eyed phenotype (C). Age of each mouse was about 30 days. Note that the black spotting mouse shows pigmented patches with a grayish tone. Bottom panels show the hair shafts from the control mouse (left), the bw mouse (middle), and the black spotting mouse (right). The hairs presented were derived from each paraffin block (insets) of the skin tissues, taken from the indicated mice of 8 weeks old.

Representative images of the mice that carry the *Dct-lacZ* transgene are shown (Fig 2A–2C): the control C57BL/6J mouse (A), the bw mouse (B), and the black spotting mouse (C). In contrast to the bw mouse that shows the completely white coat (Fig 2B), the black spotting mouse has black spots mainly on the dorsal portion and the neck region (Fig 2C). Importantly, the black spotting mouse retains the black-eyed phenotype, but the pigmented patches of the black spotting mouse show a grayish tone (Fig 2C), compared with the control C57BL/6J transgenic mouse (Fig 2A). The difference in the tone of pigmented coat color is apparent in the paraffin block of each skin tissue (insets of bottom panels in Fig 2). To explore the reason for the grayish tone of black patches of the black spotting mouse, we compared the appearance of hair shafts present in the paraffin blocks of the skin tissues (bottom panels). The hair shafts of the control mouse skin contain melanin pigments with the clearly visible frames (left), whereas the hair shafts of the bw mouse lack melanin pigments (middle). Importantly, there are two types of hairs in a black patch of the black spotting mouse: pigmented hairs and hairs lacking melanin pigments (right). Moreover, the degree of melanin deposits appeared to be lower in the pigmented hair shafts of the black spotting mouse (right), compared to the pigmented hair shafts of the control mouse (left). As expected, non-pigmented hair shafts of the black spotting mouse are indistinguishable from those of the bw mouse. Thus, the lower number of pigmented hairs may account for the grayish tone of a pigmented patch of the black spotting mouse, and a black patch of the black spotting mouse contains hair follicles lacking melanin pigments.

### Lack of follicular melanocytes in the white coat area of the black spotting mouse

**Fig 3:**
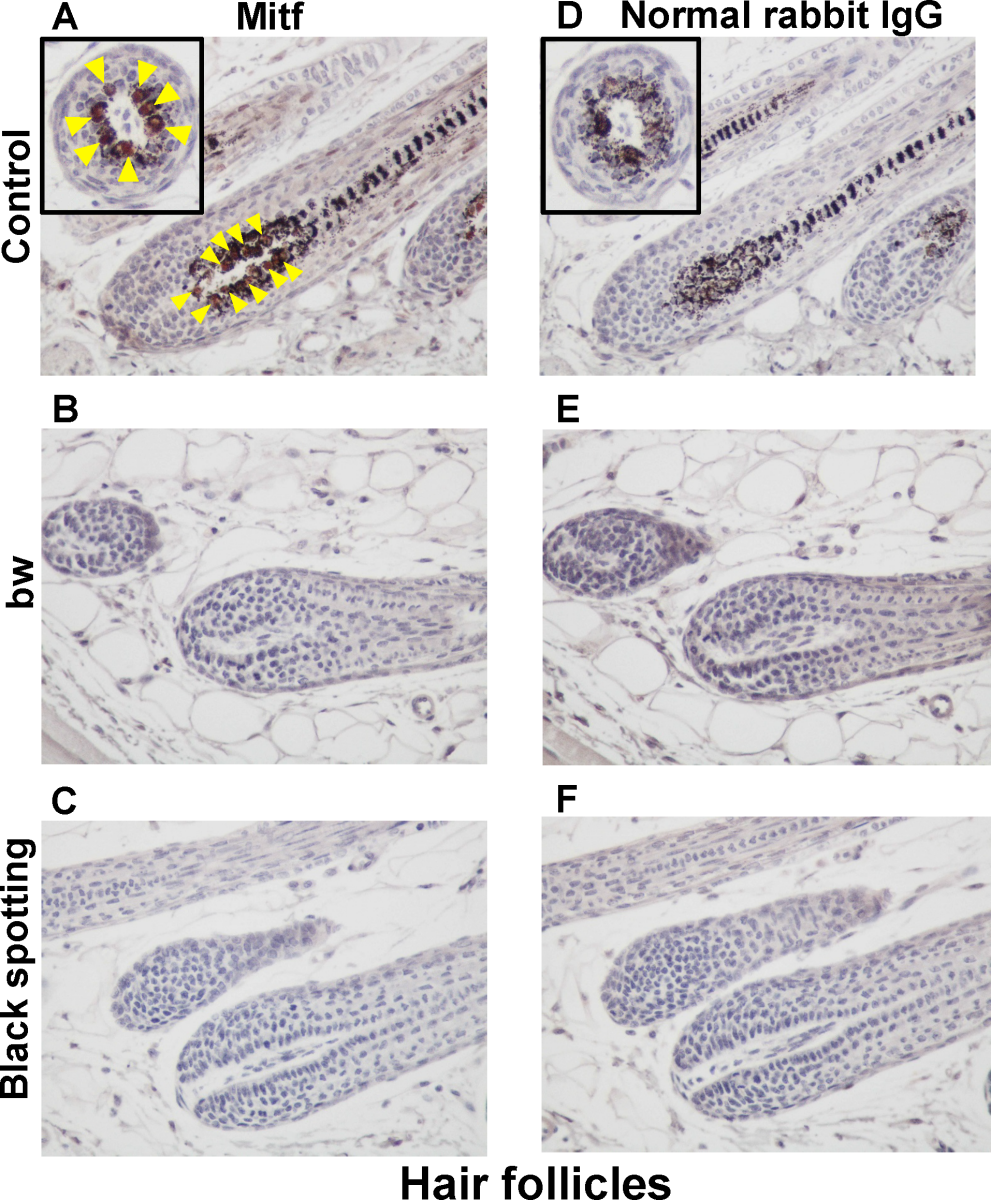
Lack of Mitf expression in the white coat area of the black spotting mouse. Immunohistochemical analysis of the hair follicles from the control lacZ transgenic mouse (A, D), from the bw mouse (B, E), and from the white coat area of the black spotting mouse (C, F). Age of each mouse was 8 weeks. The consecutive tissue section was analyzed with the anti-MITF antibody (A-C) or normal rabbit IgG as negative control (D-F) (x 400). Mitf-positive nuclei, stained as brown (arrow heads), were detected only in the hair follicles of the control lacZ transgenic mouse (A). Each inset shows the transverse section of a hair follicle (A, D).

We next immunohistochemically analyzed the Mitf expression in hair follicles at the anagen stage using the skin samples prepared from the bw mouse and the white coat area of the black spotting mouse (Fig 3). The hair follicles of the control transgenic mouse contain melanin pigments (Fig 3A and 3D), and Mitf expression, stained as brown, was detected in follicular melanocytes (arrowheads in Fig 3A). Because the brown coloration representing Mitf expression may be unclear due to the presence of melanin pigments, the transverse section of a hair follicle is also presented in inset. In contrast, melanin pigments and Mitf-positive cells were absent in the hair follicles of the bw mouse (Fig 3B and 3E). Likewise, melanin pigments and Mitf-positive cells were absent in the hair follicles at the white coat area of the black spotting mouse (Fig 3C and 3F).

**Fig 4:**
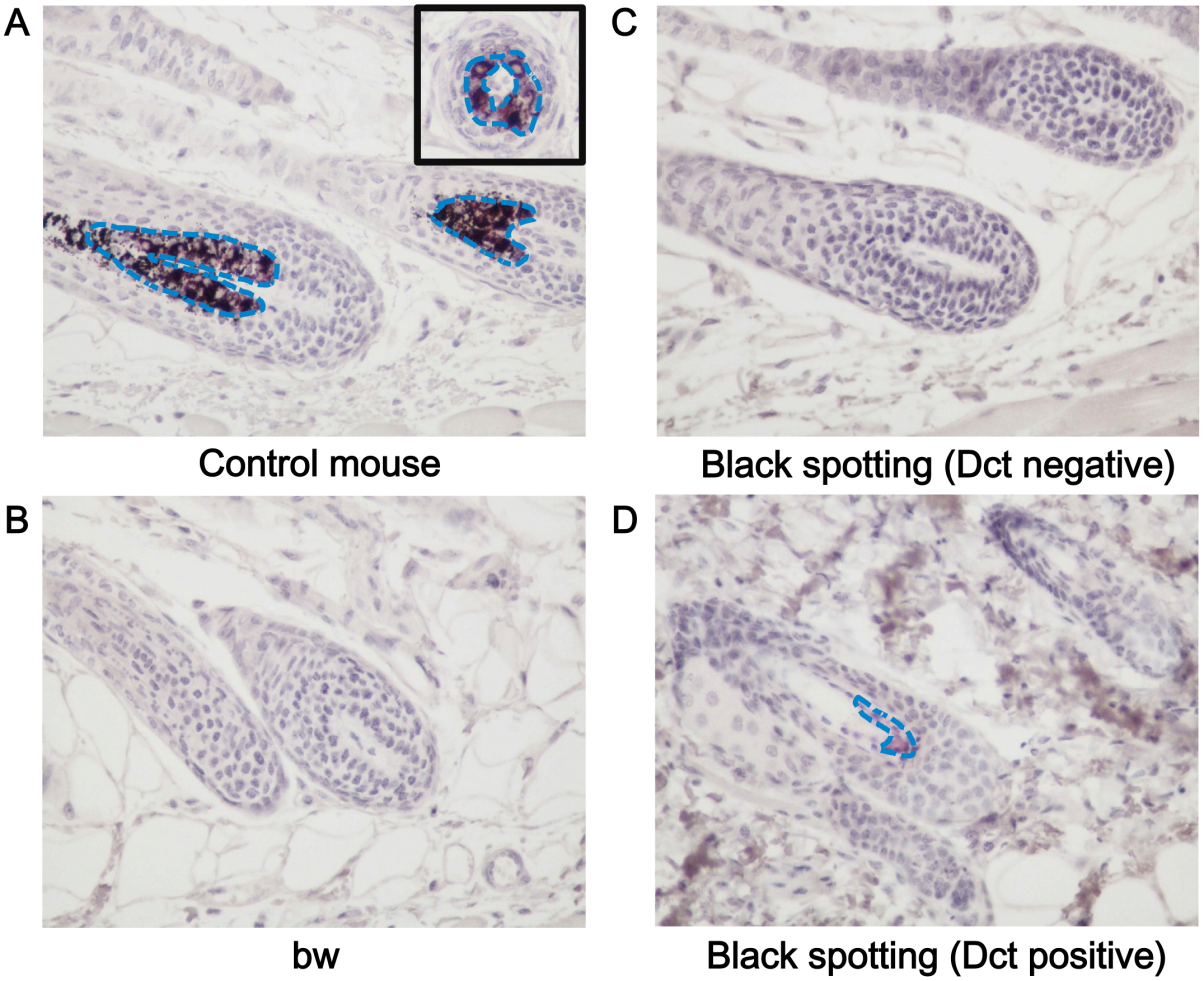
Expression of Dct in melanocytes of a pigmented hair follicle of the black spotting mouse. Immunohistochemical analysis of the hair follicles from the control lacZ transgenic mouse (A), from the bw mouse (B), and from the white coat area (C), and black coat area (D) of the black spotting mouse. Each tissue section was analyzed with the anti-Dct antibody (x 400). Dct-positive cells, stained as purple, were detected in follicular melanocytes of the control lacZ transgenic mouse (A) and in a hair follicle at the black patch of the black spotting mouse (D). An inset shows the transverse section of a pigmented hair follicle (A). Blue broken lines indicate the area where Dct-positive cells are distributed. Age of each mouse was 8 weeks.

To determine the identity of Mitf-positive cells, we next analyzed Dct expression in the hair follicles at the anagen stage by the immunohistochemical analysis (Fig 4). The Dct expression was detected in the hair follicles of the control mouse (Fig 4A), but not in the hair follicles of the bw mouse (Fig 4B) and in the hair follicles at the white coat area of the black spotting mouse (Fig 4C). Taken together, these results indicate that melanocytes are absent in the hair follicles of the bw mouse and in the hair follicles at the white coat area of the black spotting mouse. In contrast, Dct expression was detected in the hair follicles at a pigmented patch of the black spotting mouse (Fig 4D). Importantly, besides the lack of follicular melanocytes, there is no noticeable change in the structural organization of the hair follicles of the bw mouse and that of the hair follicles lacking melanocytes of the black spotting mouse.

### Retained LINE-1 insertion in the *Mitf* gene of the black spotting mouse

**Fig 5:**
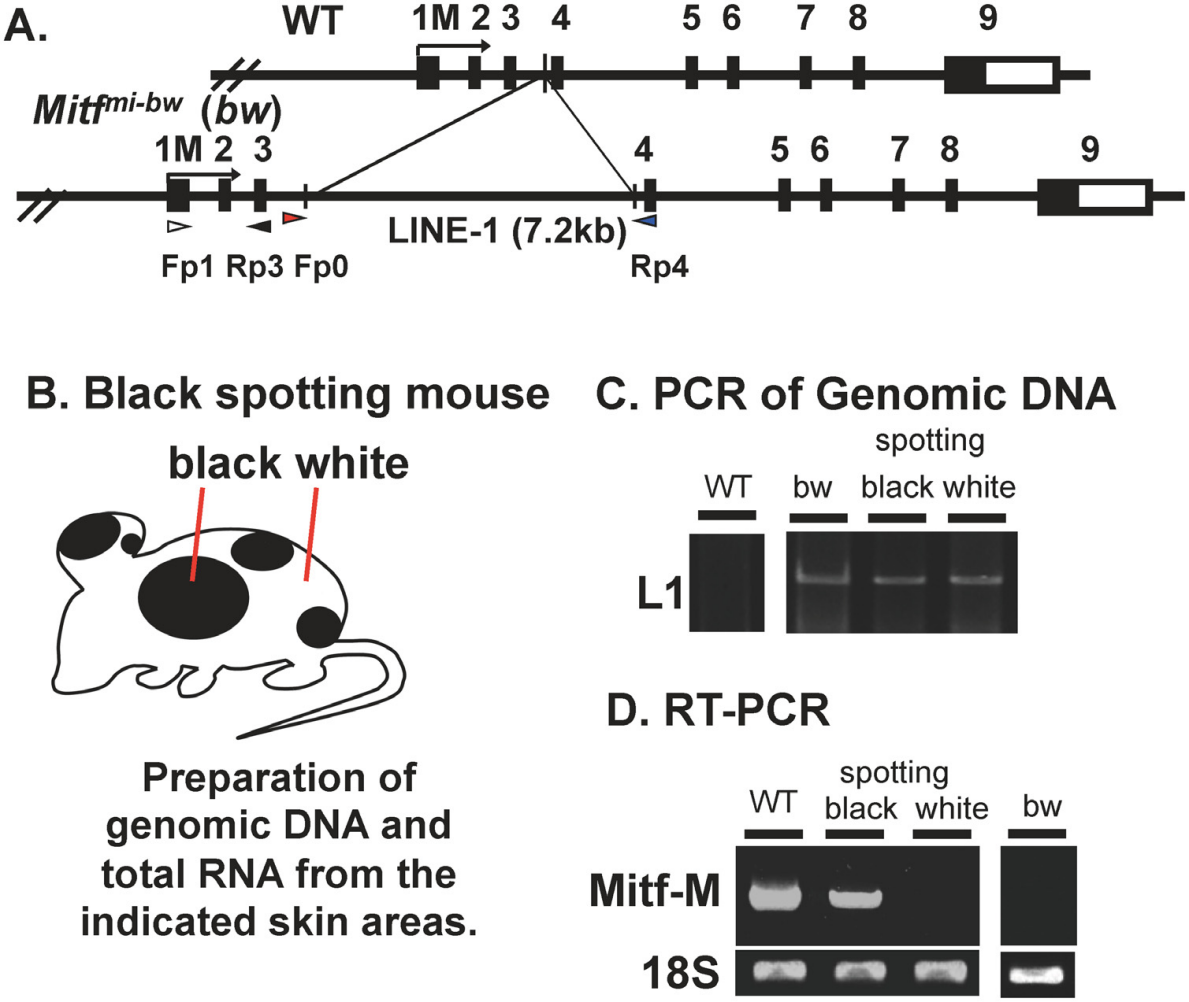
LINE-1 is retained in the Mitf gene of the black spotting mouse. (A) The wild-type *Mitf* gene (WT) and the *Mitf*^*mi-bw*^ gene are schematically presented on the top, showing the position of the LINE-1 insertion in intron 3. The promoter region upstream from exon 1M is the melanocyte–specific promoter [9]. Exon 1 M encodes the amino-terminus of Mitf-M. (B) Schematic representation of the black spotting mouse. (C) PCR of genomic DNA. A primer set (Fp0 and Rp4) was used to amplify the sequence of intron 3. Genomic DNA and total RNA were isolated from the pigmented patch and white coat area of the black-spotting mouse skin at postnatal day (P) 5.0. The age of wild-type mouse and bw mouse was 30 days. (D) RT-PCR analysis of Mitf-M gene transcripts. A primer set (Fp1 and Rp3) was used to amplify the Mitf-M transcripts. Expression of Mitf-M transcripts was detected in RNA prepared from the black patch (spotting), but was not detectable from the white coat area (white). Left lane shows positive control with skin RNA from C57BL/6J (WT). A right panel of a separate gel shows the negative control with skin RNA from bw mouse. Bottom bands are internal control (18S rRNA).

We next explored whether the LINE-1 is retained in the *Mitf* gene of the black spotting mouse (Fig 5A). Genomic DNA and total RNA were isolated from a pigmented patch or a white coat area of the black spotting mouse (Fig 5B). The PCR analysis of genomic DNA revealed the amplified products of 7.6 kb from bw mouse DNA and black spotting mouse DNA (Fig 5C), but not from wild-type mouse DNA. In the latter context, the amplified product of 460 bp from wild-type mouse DNA was detected in a separate agarose gel used (data not shown). These results exclude the possibility of the somatic reversion that eliminates the LINE-1 insertion from the *Mitf*^*mi-bw*^ gene.

We then performed the RT-PCR using skin samples taken from a pigmented patch and a white coat area of the black spotting mouse (Fig 5D), showing that Mitf-M transcripts are expressed at the black patch of the black spotting mouse as well as in the skin of the wild type mouse, which is consistent with the Dct expression in follicular melanocytes at the pigmented patch (see Fig 4D). In contrast, Mitf-M transcripts are undetectable in the white coat area of the black spotting mouse (Fig 5D) as well as in the bw mouse skin, as reported previously [8]. These results, together with the immunohistochemical data (Fig 4C), indicate the lack of follicular melanocytes in the white coat area of the black spotting mouse.

### Attenuated expression of beta-catenin and cyclin D1 in the hair follicles lacking melanocytes

**Fig 6:**
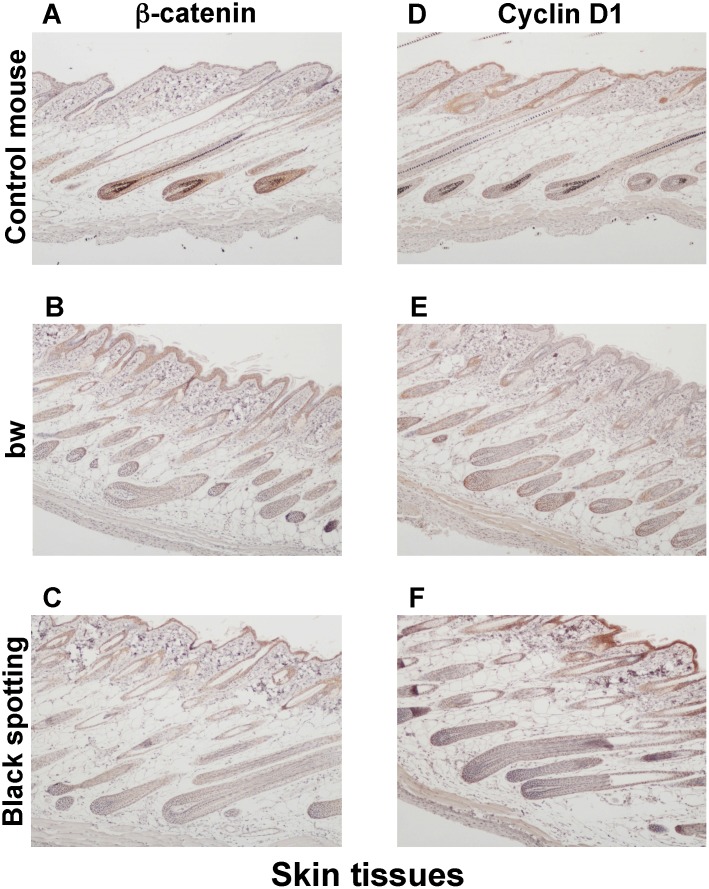
Expression of beta-catenin and cyclin D1 in the skin tissues. Immunohistochemical analysis of the skin tissues taken from the control transgenic mouse (A, D), the bw mouse (B, E), and the black spotting mouse (C, F) for expression of beta-catenin (A—C) and cyclin D1 (D—F) (x 100). The age of mice analyzed was 8 weeks. The expression of beta-catenin and cyclin D1 was detected in the hair follicles of the control mouse. The intensity for the expression of cyclin D1 appears highest in the epidermis of the black spotting mouse (F).

As a first step to explore the functional consequence of the LINE-1 insertion that may determine the fate of skin melanocytes, we analyzed the expression profiles of beta-catenin and cyclin D1 in the skin tissues, isolated from the control transgenic mouse, the bw mouse, and the black spotting mouse (Fig 6). Beta -catenin is a downstream component of Wnt signaling [19], the signal of which is actively operating in the hair shaft precursor cells [20] and melanocytes [21]. Cyclin D1 is a target of Wnt/beta-catenin signaling. Furthermore, beta-catenin may function as a coactivator for MITF in melanocytes [22, 23]. Overall, the appearance of the skin tissues was similar among the control transgenic mouse (Fig 6A and 6D), the bw mouse (Fig 6B and 6E), and the black spotting mouse (Fig 6C and 6F), except for the thickened epidermal layer of the bw mouse. Compared with the control mouse (Fig 6A and 6D), the signal intensity for the expression of beta-catenin and cyclin D1 was lower in the hair follicles of bw mouse (Fig 6B and 6E) and the black spotting mouse (Fig 6C and 6F). In contrast, the staining for beta-catenin was apparent in the epidermis of the bw mouse and the black spotting mouse, compared to the control mouse epidermis (Fig 6A–6C). Moreover, the staining intensity for the cyclin D1 expression was higher in the keratinocyte layer of the black spotting mouse (Fig 6F), compared to the control mouse (Fig 6D) and the bw mouse (Fig 6E).

**Fig 7:**
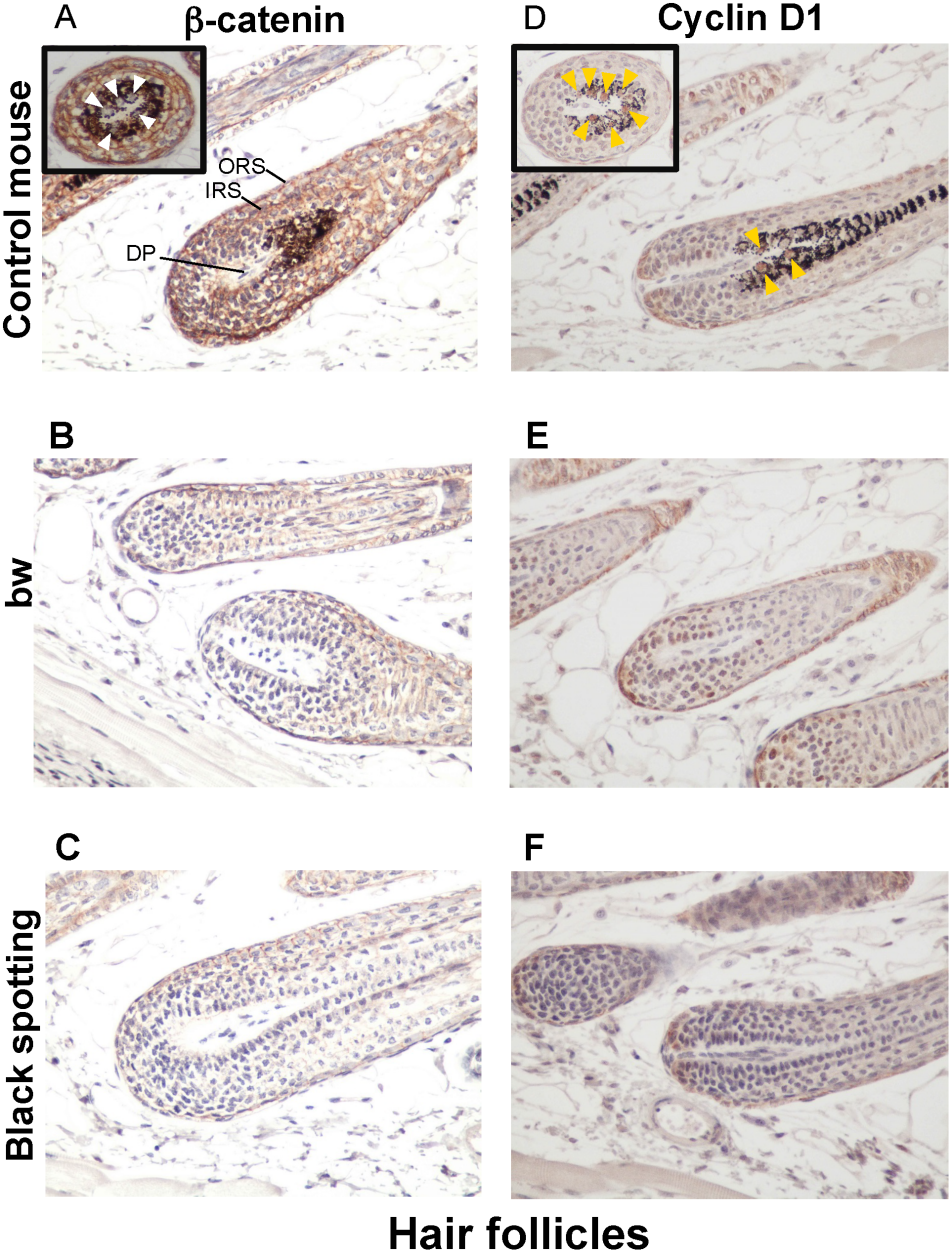
Low expression of beta-catenin and cyclin D1 in hair follicles lacking melanocytes. Immunohistochemical analysis of the hair follicles taken from the control lacZ transgenic mouse (A, D), the bw mouse (B, E), and the black spotting mouse (C, F) for expression of beta-catenin (A—C) and cyclin D1 (D—F) (x 400). Each inset shows the transverse section of a pigmented hair follicle (A and D). In panel A, the signal for beta-catenin was detected in membranes but not in nuclei (white arrowheads), while in panel D, the cyclin D1 was expressed in nuclei (yellow arrowheads). The tissue sections shown were adjacent to the tissue sections used in Fig 6. DP, dermal papilla; ORS, outer root sheath; and IRS, inner root sheath.

Consequently, using larger magnification of the skin tissue sections (x 400), we compared the expression profiles of beta-catenin and cyclin D1 in the hair follicles of the control transgenic mouse (Fig 7A and 7D), the bw mouse (Fig 7B and 7E), and the black spotting mouse (Fig 7C and 7F). The expression of beta-catenin was detected in follicular cells, located around the dermal papilla and in the outer root sheath and the inner root sheath (Fig 7A); namely, beta-catenin was expressed in matrix cells around the dermal papilla, melanocytes, and the precursor cells for the cortex of hair shaft. However, the nuclear localization of beta-catenin was not clear in follicular melanocytes of the control mouse (white arrowheads in Fig 7A), suggesting that the hair follicles analyzed were at later anlagen stage. In contrast, the staining intensity for beta-catenin expression was apparently lower in the hair follicles lacking melanocytes of the bw mouse (Fig 7B) and the black spotting mouse (Fig 7C), compared to the control mouse (Fig 7A). Moreover, the signal intensity for beta-catenin was similar in hair follicles between the bw mouse (Fig 7B) and the black spotting mouse (Fig 7C). Importantly, the cyclin D1 expression was detected in the nuclei of follicular melanocytes of the control mouse (yellow arrowheads in Fig 7D), suggesting that Wnt signaling may be active in follicular melanocytes. The cyclin D1 expression was similar in hair follicles between the bw mouse (Fig 7E) and the black spotting mouse (Fig 7F). These results suggest that follicular melanocytes may support the expression of beta-catenin in their surrounding cells, such as matrix cells.

### Enhanced expression of beta-catenin and cyclin D1 in the epidermis of the black spotting mouse

**Fig 8:**
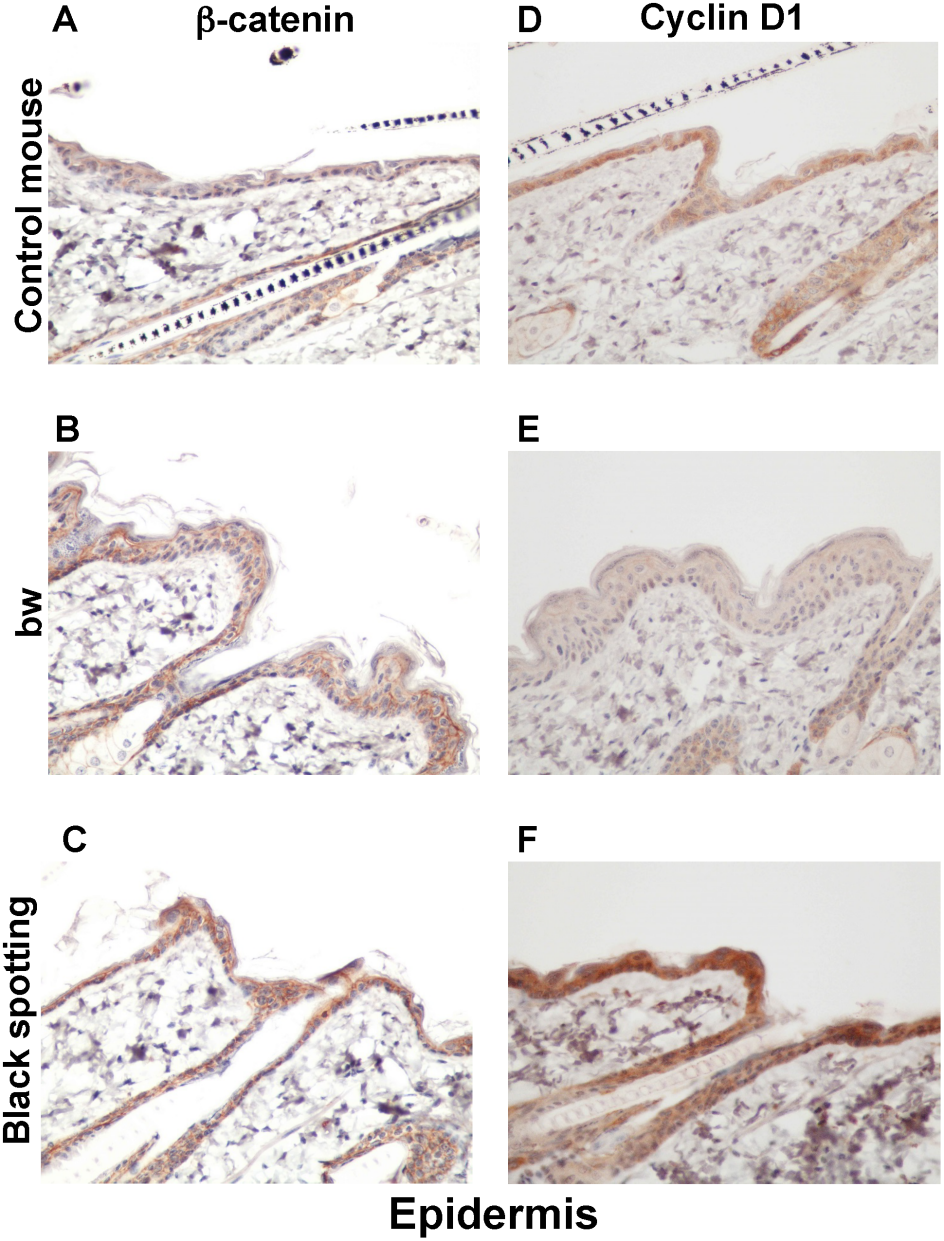
Expression of beta-catenin and cyclin D1 in the epidermis of the black spotting mouse. Immunohistochemical analysis of the epidermal regions taken from the control transgenic mouse (A, D), the bw mouse (B, E), and the white coat area of the black spotting mouse (C, F) for expression of beta-catenin (A—C) and cyclin D1 (D—F) (x 400). Shown are the images of the epidermal portions of the same tissue sections used in Fig 7. The epidermal thickening is apparent in the bw mouse.

We next compared the epidermal expression of beta-catenin and cyclin D1 among the control mouse (Fig 8A and 8D), the bw mouse (Fig 8B and 8E), and the black spotting mouse (Fig 8C and 8F). The images shown represent the epidermal portions of the same tissue sections used in Fig 7. Apparently, the staining intensity for beta-catenin was higher in the epidermis of the black spotting mouse, compared to the bw mouse and the control mouse epidermis (Fig 8A–8C). Moreover, the signals for beta-catenin appeared to be localized in the membrane region of keratinocytes in the bw mouse epidermis (Fig 8B). In this context, the staining intensity for cyclin D1 was lower in the bw mouse epidermis (Fig 8E), compared to that in the control mouse epidermis (Fig 8D) and the black-spotting mouse epidermis (Fig 8F). Importantly, the staining intensity for beta-catenin and cyclin D1 was highest in the epidermis of the black spotting mouse (Fig 8C and 8F). We also confirmed that the keratinocyte layer was thicker in the bw mouse (Fig 8B and 8E), compared to the control mouse (Fig 8A and 8D) and the black spotting mouse (Fig 8C and 8F). These results suggest that beta-catenin may not properly function in keratinocyte nuclei of the bw mouse, whereas the function of beta-catenin may be enhanced in keratinocytes of the black spotting mouse.

### Enhanced expression of Ki-67 in the basal layer of the bw mouse epidermis

**Fig 9:**
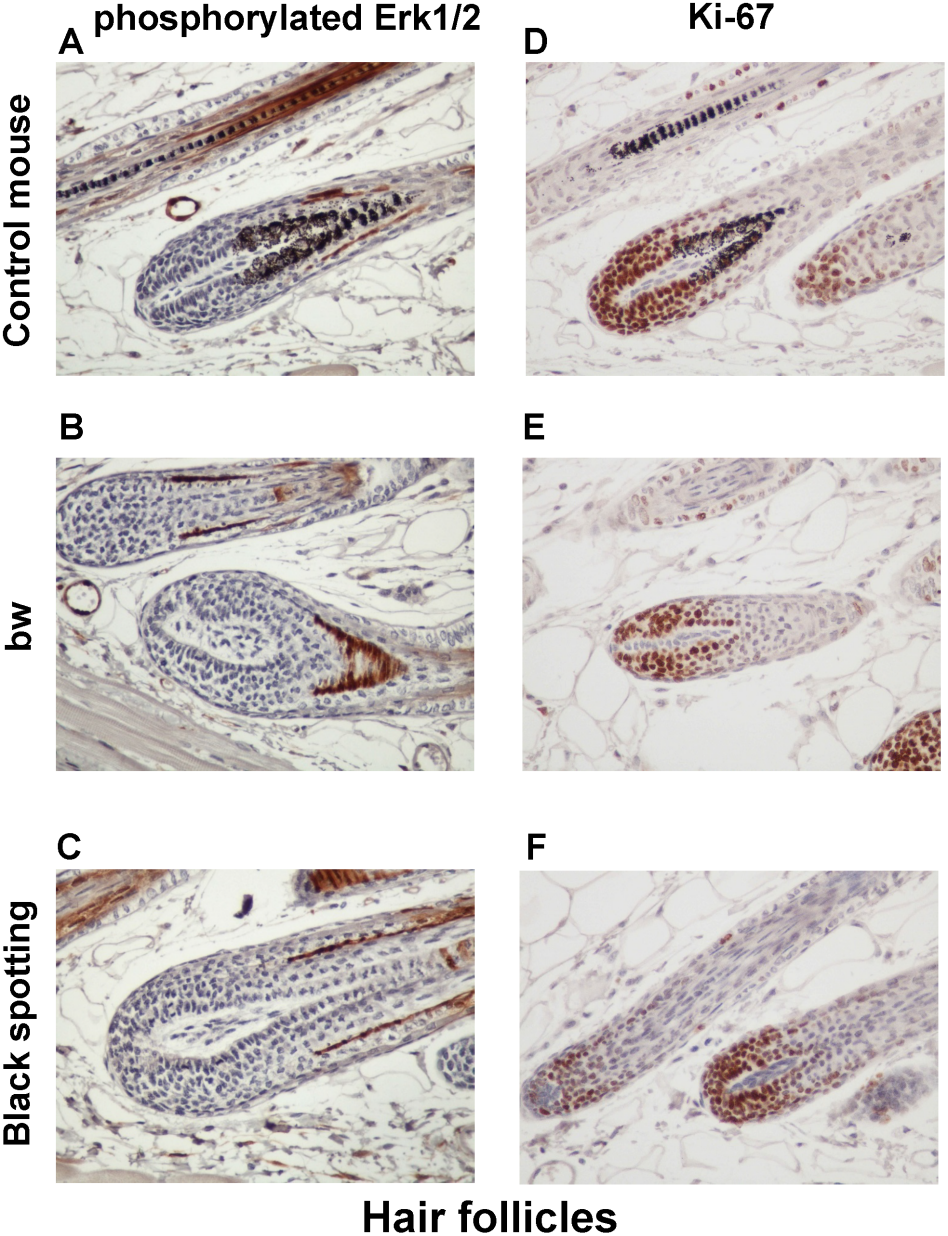
Expression of phosphorylated ERK1/2 and Ki-67 in hair follicles. Immunohistochemical analysis of the hair follicles of the control transgenic mouse (A, D), the bw mouse (B, E), and the black spotting mouse (C, F) for expression of p-ERK1/2 (A—C) and Ki-67 (D—F) (x 400). Shown are the images of the adjacent tissue sections used in Fig 7.

**Fig 10:**
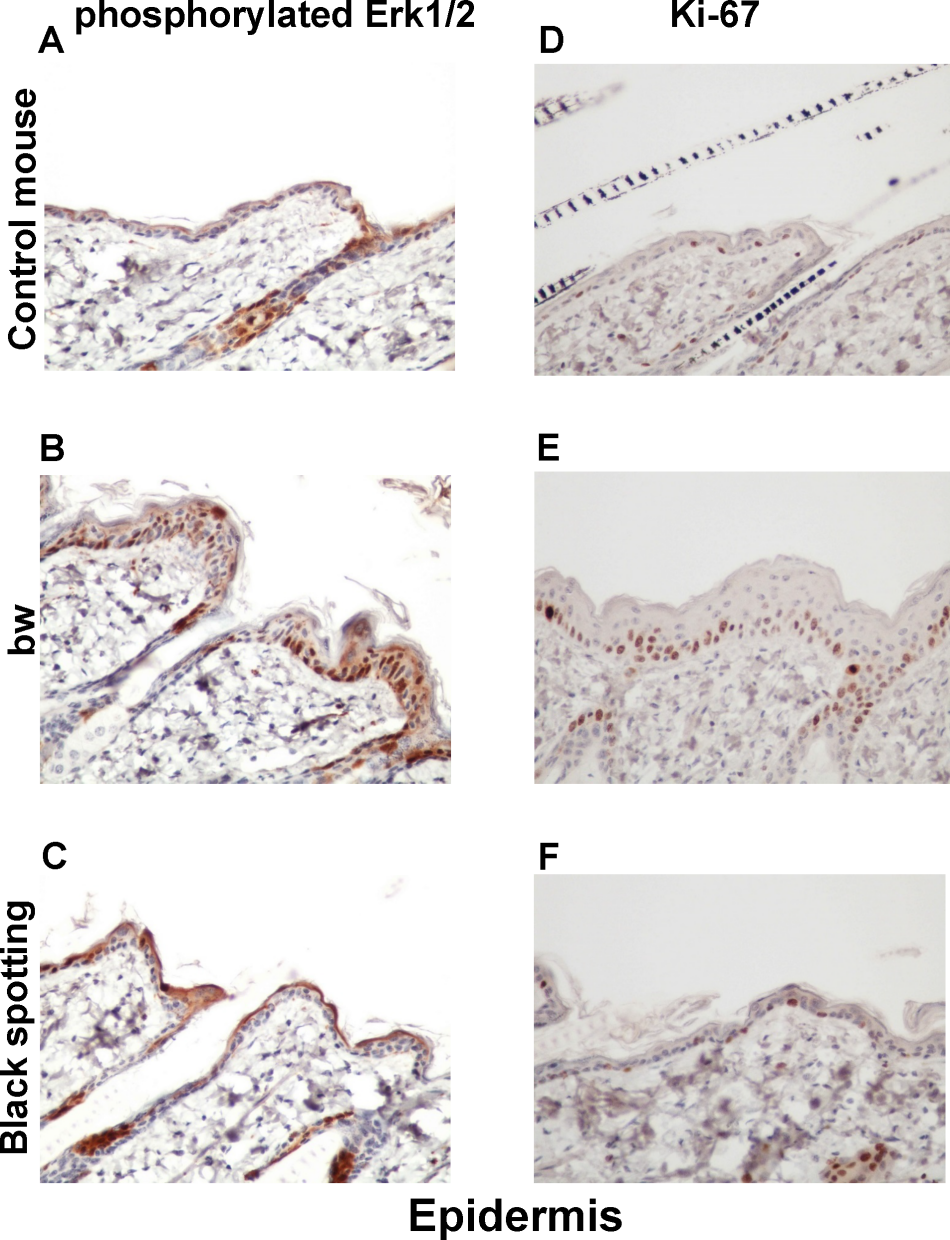
Expression of phosphorylated ERK1/2 and Ki-67 in the epidermis. Immunohistochemical analysis of the epidermal regions taken from the control transgenic mouse (A, D), the bw mouse (B, E), and the white coat area of the black spotting mouse (C, F) for expression of p-ERK1/2 (A—C) and Ki-67 (D—F) (x 400). Shown are the images of the epidermal portion of the same tissue sections used in Fig 9.

To explore the molecular basis of the thickened keratinocyte layer in the bw mouse, we next analyzed the expression of p-ERK1/2 and Ki-67 in hair follicles (Fig 9) and the epidermis (Fig 10) of the consecutive skin tissue sections. The expression of p-ERK1/2 and Ki-67 was similar in hair follicles among the three mouse groups (Fig 9). Especially, Ki-67 expression was detected in matrix cells surrounding the dermal papilla. In contrast, the staining intensity for Ki-67 was higher in the basal layer of the bw mouse epidermis (Fig 10E), compared to the control mouse and the black spotting mouse (Fig 10D and 10F). Importantly, the epidermal expression of p-ERK1/2 and Ki-67 was similar in the control mouse and the black spotting mouse (Fig 10). The enhanced expression of Ki-67 may reflect the thickening of the keratinocyte layer of the bw mouse. We therefore suggest that the epidermal microenvironment of the black spotting mouse may be suitable for normal growth of keratinocytes, which may contribute to the survival of developing melanoblasts in the selected areas.

### Formation of presumptive black spots by E15.5 in the black spotting mouse

**Fig 11:**
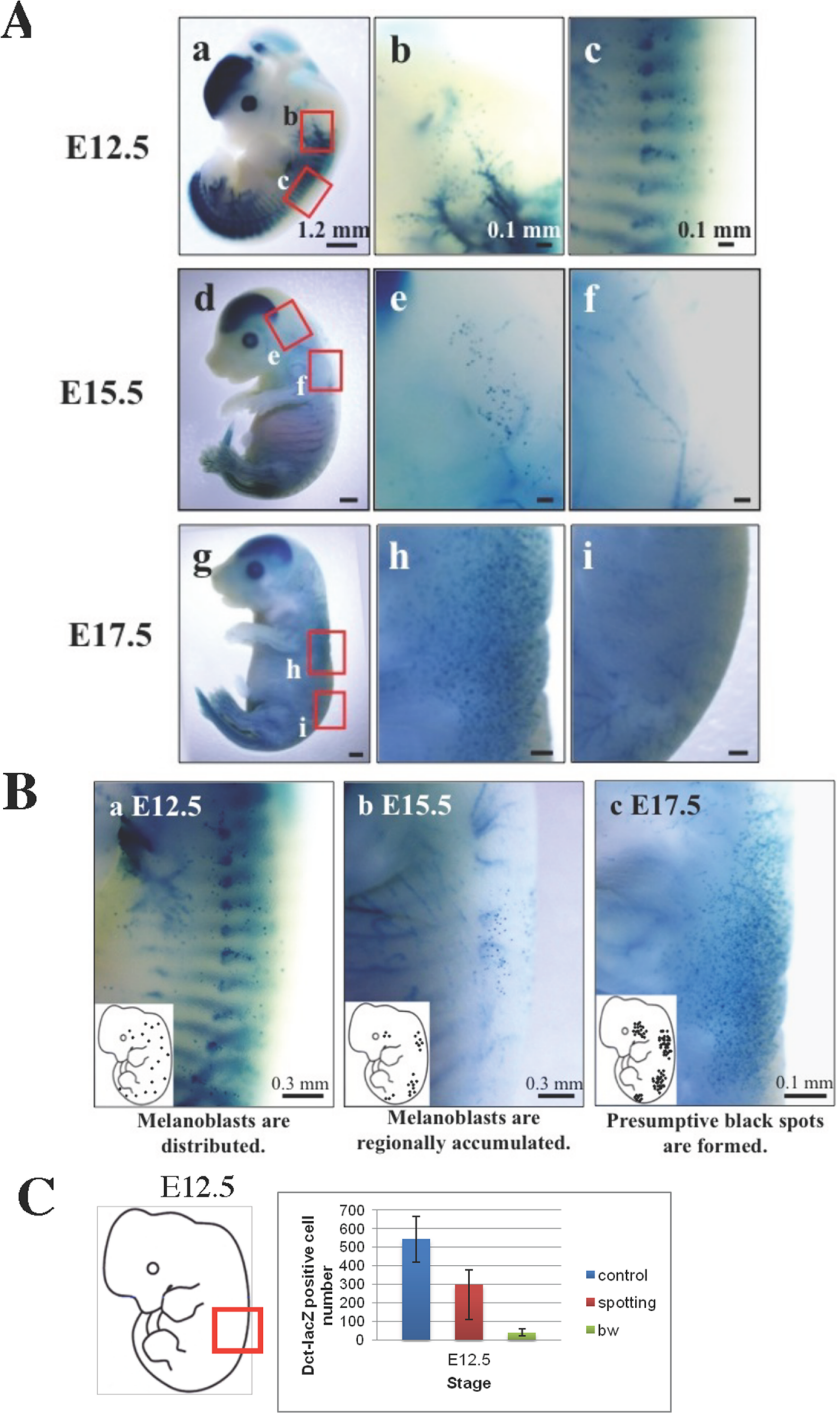
Formation of presumptive black patches in the black spotting mouse by E15.5. A. Shown are the X-gal staining images of homozygous black-spotting mouse embryos at the indicated embryonic days. The areas indicated with red square are enlarged and shown at right. At E12.5, melanoblasts are widely distributed in black spotting mice (a, b, c). At E15.5, melanoblasts are regionally accumulated (d). They are present (e) or absent (f), depending on the areas. At E17.5, sharply defined accumulation of melanoblasts is apparent (g, h), but melanoblasts are not present in other area (i). Scale bars: 1.2 mm in (a), (d) and (g), and 0.1 mm in others. B. A representative dorsal region is shown for each embryonic day. Inset shows schematic representation for the distribution of surviving melanoblasts, with the emphasis on the appearance of presumptive black patches at E17.5. Note that the drawing of each embryo does not reflect the actual image of the embryo at the indicated age. C. Difference in the number of X-gal-stained cells among C57BL/6J transgenic mouse embryos (control), black spotting mouse embryos (spotting), and bw mouse embryos at E12.5. The area used for cell counting is schematically shown with red square. Note that the tissue sections containing many Dct-lacZ-positive cells were selected from the black spotting mouse embryos at E12.5, whereas every tissue section contained a large number of Dct-lacZ-positive cells from control mouse embryos at E12.5 [5]. The Dct-lacZ-positive cell number/counted area is presented as mean ± S.D. of at least three embryos, although the data were obtained with a semi-quantitative measure. The data of control mouse embryos and bw mouse embryos were taken from the published paper [5] and are shown for comparison. At E12.5, the number of developing melanoblasts was consistently lower in the black spotting mouse than that in control mouse.

To explore the timing of the formation of the black spots, we took the advantage that the black spotting mouse carries the *Dct-lacZ* transgene. The number and the distribution of *Dct*-*lacZ*-positive melanoblasts, stained blue, were analyzed during embryonic development of the black spotting mouse (Fig 11A and 11B). In addition to the ectopic expression of the transgene in caudal nerves [24, 25], the RPE and the telencephalon, where the endogenous *Dct* gene is expressed [26], were stained blue. At E12.5, *Dct-lacZ*-positive melanoblasts were easily detected in the trunk region of the black spotting mouse embryo (Fig 11A and 11B), although the density of the *Dct-lacZ*-positive cells was apparently lower in the black spotting mouse embryo, compared to the control mouse embryo [5]. In fact, the number of the *Dct-lacZ*-positive cells per counted area was consistently lower in the black spotting mouse embryo than that in the control mouse embryo (Fig 11C). In contrast, at this stage, the *Dct-lacZ*-positive cells were hardly detected in the bw mouse [5]. Thus, the migration ability of neural crest cells is better retained in the black spotting mouse, compared to the bw mouse.

Subsequently, at E15.5 (Fig 11A and 11B), the *D*ct-*lacZ*-positive cells accumulated in restricted areas, while the *Dct-lacZ*-positive cells were undetectable in other areas. In other words, certain groups of melanoblasts survived in the presumptive pigmented patches, and other groups of melanoblasts disappeared in the presumptive white coat areas. Thus, the fate of developing melanoblasts is determined by E15.5 in the black spotting mouse. In contrast, the *D*ct-*lacZ*-positive cells disappeared by E13.5 in the bw mouse [5]. Such a difference in the fate of developing melanoblasts may reflect the fluctuation in the functional consequence of the LINE-1 insertion. At E17.5, *Dct-lacZ*-positive melanoblasts were easily detected as a cluster at the presumptive pigmented patches (Fig 11B). These results suggest that melanoblasts can proliferate and differentiate into melanocytes in certain restricted areas of the black spotting mouse, despite the LINE-1 insertion in its *Mitf* gene.

### Aberrantly spliced Mitf-M transcripts expressed in the pigmented patches

**Fig 12:**
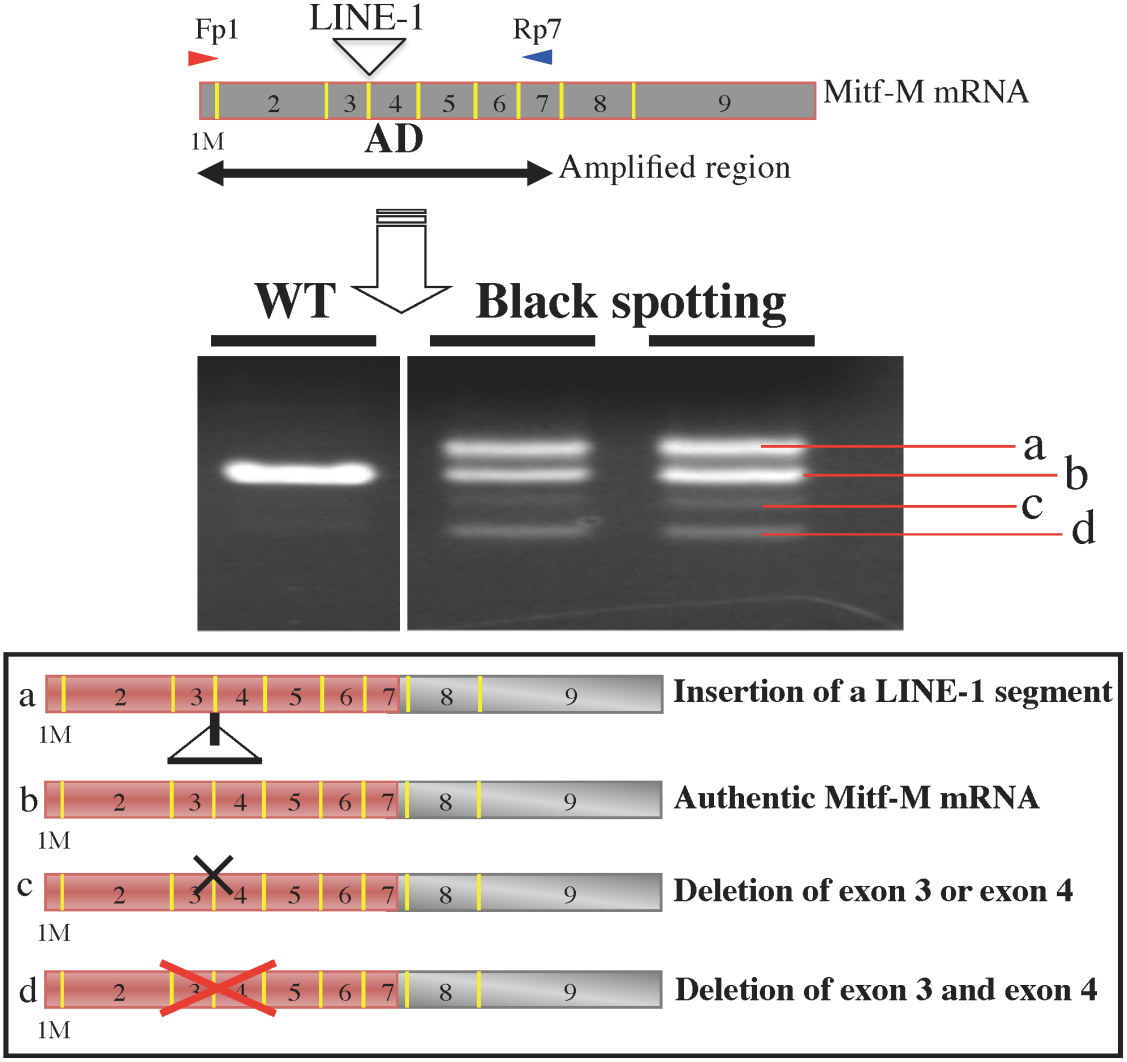
Expression of aberrantly spliced Mitf-M transcripts at black patches of the black spotting mouse. The RT-PCR analysis was performed with the downstream reverse primer on exon 7 (Rp7). RNA was isolated from the skin of each mouse at the age of 30 days (P30). Exon 4 encodes an activation domain (AD). PCR products were detected as a single band with RNA from wild type mouse skin (WT). In two separate black patches from the black spotting mouse (P30), aberrantly spliced Mitf-M transcripts were detected in addition to authentic Mitf-M mRNA. The sizes of four major PCR products are 882 bp (product a), 778 bp (product b), 694 bp or 682 bp (product c), and 598 bp (product d). The product c contained two species of PCR products: the 694-bp band lacking exon 3 and the 682-bp band lacking exon 4. The predicted identity of the splicing products is shown at bottom: (a) Mitf-M mRNA containing a portion of LINE-1 (104-bp segment), (b) authentic Mitf-M mRNA, (c) Mitf-M mRNA lacking exon 3 or exon 4, and (d) Mitf-M mRNA lacking exon 3 and exon 4. The amplified region is shown in red.

To explore the molecular basis for the formation of the black spotting phenotype, we performed the RT-PCR analysis using other primer set: exon-1M forward primer (Fp1) and exon-7 reverse primer (Rp7) (Fig 12). With this primer set, we were able to detect the aberrantly spliced Mitf-M transcripts related to the LINE-1 insertion [8]. In the wild-type mouse skin, the PCR products were detected as a single band that represents authentic Mitf-M mRNA, whereas multiple PCR products were detected in the skin samples taken from the separated pigmented patches of the black spotting mouse (Fig 12). Sequencing analysis revealed that these PCR products represent (a) Mitf-M mRNA containing a LINE-1 segment of 104 bp, (b) authentic Mitf-M mRNA, (c) Mitf-M mRNA lacking exon 3 or exon 4, and (d) Mitf-M mRNA lacking both exon 3 and exon 4. These splicing products were identical to those detected in the bw mouse brain [8]. These data reconfirm that the LINE-1 insertion is retained in the *Mitf* gene of the black spotting mouse, thereby excluding the possibility of the somatic reversion. Importantly, the Mitf-M mRNA lacking the exon-4 sequence was shown to encode a dominant-negative form of Mitf-M protein [8], because exon 4 encodes the transactivation domain of Mitf [27]. Thus, the LINE-1 insertion may result in the decrease in the level of functional Mitf-M in melanoblasts and melanocytes at the pigmented patches of the black spotting mouse, which may also account for the grayish tone of pigmented patches (see Fig 2). However, the Mitf-M deficiency does not impair the development of melanoblasts in restricted areas of the black spotting mouse.

### Age-related graying of pigmented patches

**Fig 13:**
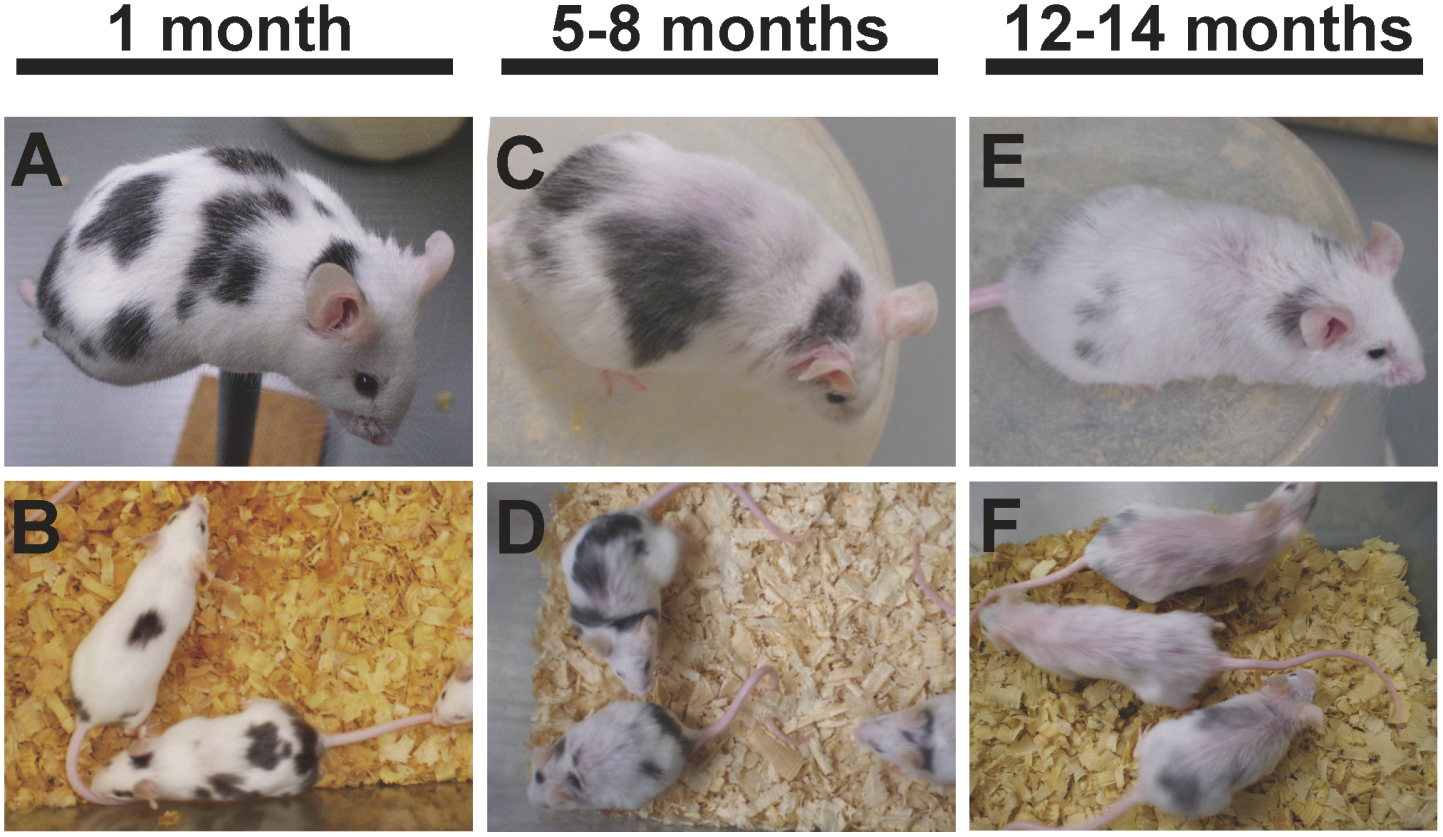
Age-dependent graying of pigmented hairs in the black patches. The black patches get gray with age. At 1 month after birth, black spotting mice have distinct black patches (A and B). By 5–8 months, the mice get more gray hairs (C and D), and by 12 months, most of hairs in the black patches turned white (E and F).

As described above, the black spotting mouse may provide a good model to study the phenotypic consequence of the Mitf-M deficiency, prompting us to analyze the age-related changes of black spots. As expected, the black spotting mouse shows apparent graying (Fig 13). By about 6 months after birth, graying was noticeable, and by about 12 months, almost all hairs of the black spots turn to gray or white. These results suggest that melanocyte stem cells or melanocytes in the hair follicles at the black patches may be more vulnerable to certain aging-related events and thus these cells fail to survive. We have therefore proposed that the Mitf-M deficiency may lead to premature death of melanocyte stem cells or melanocytes in the hair follicles of the black spotting mouse. Alternatively, the phenotypic consequence of the LINE-1 insertion may vary with age.

### Diluting effect of the *Mitf*^*mi-bw*^ allele on the black spotting phenotype

**Fig 14:**
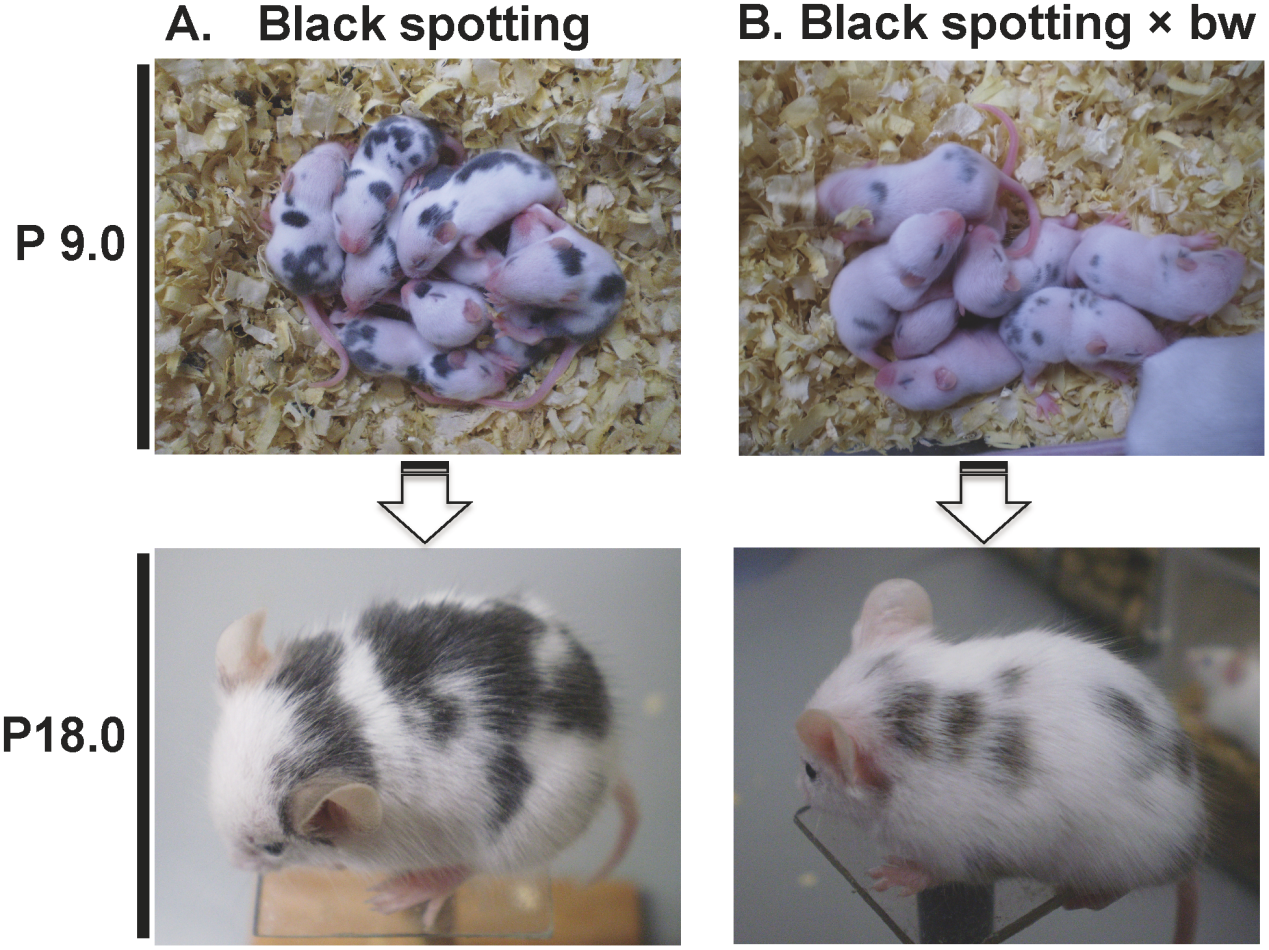
Diluting effect of the *Mitf*^*mi-bw*^ allele on the black spotting phenotype. Shown are the black spotting mice at postnatal day (P) 9.0 (A) and the offspring at P 9.0 of male black spotting mouse × female bw mouse (B). There is apparent difference in the color of the black spots between black spotting mice and (black spotting × bw) mice. Each representative mouse is shown at bottom. At P 18.0, the difference became clear; the black spotting offspring (left) has larger black patches, compared to the black patches of the offspring of black spotting mouse × bw mouse (right). In addition, the mouse of black spotting × bw has small black patches (right).

To explore the inheritance of the black spotting phenotype, we crossed a male black spotting mouse with a female bw mouse or a female black spotting mouse with a male bw mouse. Irrespective of the combination, all the offspring of black spotting mice mated with bw mice have pigmented patches with diluted color, compared to those of black spotting mice (Fig 14A and 14B). In addition, the size of pigmented patches tends to be smaller in the heterozygous black spotting mouse with bw mouse, compared to the patches seen in the homozygous offspring of the black spotting mouse (Fig 14, bottom panels). Thus, the black spotting phenotype is diluted in the presence of the *Mitf*^*mi-bw*^ allele; namely, the number of pigmented hair follicles is decreased in the offspring of the black spotting mouse mated with the bw mouse. We also crossed the black spotting mouse with the C57BL/6 mouse, yielding the offspring with black coat color that is indistinguishable from the C57BL/6 mouse (photos not shown). In conclusion, the black spotting phenotype is inherited in a semi-dominant manner for the bw phenotype, whereas the black spotting phenotype appears to be inherited as a recessive trait for the white-coat phenotype.

## Discussion

### Features of the grayish tone of pigmented hairs

The presence of non-pigmented hairs is responsible in part for the grayish tone of a black patch of the black spotting mouse (Fig 2), which may reflect the lower number of developing melanoblasts in the trunk region of the black-spotting mouse embryo at E12.5 (about 50% reduction), compared to the control mouse embryo (Fig 11C). It is therefore conceivable that follicular melanocytes may also decrease in their number even at a given black patch. In addition, the amount of functional Mitf-M protein may be decreased in follicular melanocytes at a pigmented patch, because of the expression of aberrantly spliced Mitf-M transcripts, including the transcript encoding a dominant negative isoform lacking exon 4 (the activation domain) [8]. Taken together, these findings suggest that melanin production may be decreased in follicular melanocytes at a black patch of the black spotting mouse.

The early graying seen in the black spotting mouse is reminiscent of a recessive vitiligo mouse carrying the *Mitf*^*vit*^ allele [28]. The *Mitf*^*vit*^ mouse was spontaneously arisen in the C57BL/6J strain and is characterized by the congenital white spotting on its black coat background, associated with the age-related graying of the pigmented hairs [28]. In contrast, the black spotting phenotype was originally arisen in the bw mouse on the mixed C3;B6 background [2]. The *Mitf*^*vit*^ gene carries a single-base change that results in the Asp-to-Asn substitution at position 222 in the basic helix-loop-helix region [29], and the *Mitf*^*vit*^ mutation is expected to alter the function of all Mitf isoforms. Indeed, the *Mitf*^*vit*^ mutation appears to impair the cooperation with Lef-1 or other factors [22]. Thus, the pathogenesis for early graying of the *Mitf*^*vit*^ mouse is the Mitf deficiency, which is essentially similar to that of the black spotting mouse.

### Molecular basis of the black spotting phenotype

In this study, we are unable to clarify the molecular basis for the black spotting phenotype; namely, it remains to be investigated why the black spotting mouse could partly rescue melanoblasts during embryonic development. One possibility is that the black spotting mouse carries a mutation probably in a modifier gene that may support Mitf expression or attenuate the influence of L1 insertion on Mitf expression in developing melanoblasts. In this context, we have shown that the forced expression of Mitf-M in cultured neural tube isolated from E9.5 bw embryos increases the number of migrating neural crest cells and enhances the differentiation of migrating melanoblasts [5]. Thus, the Mitf-M deficiency associated with the LINE-1 insertion reduces the migration of neural crest cells from the neural tube and the differentiation of migrating melanoblasts. In fact, the expression level of Mitf-M mRNA is lower in bw mouse embryos than that in control mouse embryos at E11.5, and subsequently, it was undetectable at E13.5 in bw mice [5]. Moreover, we have shown the enhanced apoptosis of bw melanoblasts during embryonic development [5]. These results indicate that the critical level of Mitf-M is required for the survival of melanoblasts around E13.5. Thus, a certain population of melanoblasts can survive at E13.5 probably due to the increase in Mitf-M function of the black spotting mouse. The relevant mutation may assure the activity of Mitf-M that is very close to the threshold level for the melanoblast survival.

Moreover, we have shown the apparent differences in the epidermal phenotype between the bw mouse and the black spotting mouse (Figs 8 and 10). The bw mouse shows the thickened keratinocyte layer with the higher expression of Ki-67 and the lower expression of cyclin D1, compared to the black spotting mouse. In fact, the nuclear expression of beta-catenin was not apparent in the bw keratinocytes (Fig 8). In other words, the epidermal features of the black spotting mouse are similar to those of the control mouse. Thus, a gene mutation responsible for the black spotting phenotype may also influence the epidermal development. Alternatively, a certain environmental factor may cause an epigenetic change, such as histone modification, in the Mitf gene with L1 insertion. Further analyses are required, such as the sequencing the LINE-1 present in the black patches and high-density SNP genotyping. We would like to leave these issues for the future.

On the other hand, we have shown that the immunoreactive Mitf is expressed in projection neurons, mitral cells and tufted cells, of the wild-type mouse olfactory bulb, and the distribution of these Mitf-expressing neurons was similar to that found in the bw mouse and the control transgenic mouse [13]. Moreover, the real-time RT-PCR analysis revealed the expression of Mitf-M mRNA in the wild-type mouse olfactory bulb as well as in the bw mouse olfactory bulb, but not in the bw mouse eyeball [13]. These results suggest the regional difference in the functional consequence of the LINE-1 insertion between certain neurons and melanocytes located in the skin [5], inner ear [14, 30] and retinal choroid [2]. Thus, understanding the molecular basis for the neuron-specific expression of Mitf will help us to identify the responsible gene for the black spotting phenotype.

### Implications of the black spotting mouse

We have shown that follicular melanocytes are responsible for maintaining the expression of beta-catenin in the hair follicle as well as for the keratinocyte homeostasis. Thus, the present study has provided evidence for the link between the epidermal microenvironment and melanocyte development/survival. Moreover, the presence of the black spotting mouse indicates the regional difference in the epidermal environment that may determine the Mitf-M expression level and the fate of melanocytes. Thus, the black spotting mouse provides a model system to study the molecular basis for dynamic changes in the functional consequence of the LINE-1 insertion. Lastly, the present study suggests the possibility that human diseases associated with LINE-1 insertion may present the phenotypic variability.
